# Examining in the Department of the Interior

**Published:** 1881-12

**Authors:** 


### Examining into the Department of the Interior.

The aggressive nature of surgery is being continually shown by its encroachments upon the domain of medicine, and each year some new advance of this kind indicates the progress of our art. The laryngoscope, the rhinoscope and the otoscope have revealed to us regions almost unknown, and the ophthalmoscope especially, opened to our eyes a field not only undiscovered, but one whose beauty and importance was entirely unsuspected. These, with similar instruments of precision, are most acceptable aids to study, and their value has been tested by long experience. But when we read of various appliances, by means of which the surgeon can survey with ease the mysterious depths of the urethra, or, by properly manipulating a peculiar speculum, he can placidly gaze far beyond the sigmoid flexure almost up to the coecal valve,—then does the spirit of skepticism steal in upon us, and without any play upon words, we speak of these as extraordinary tales. And now of late still another item has been going the rounds of the press in relation to a certain gastroscope invented by Leiter, the Vienna instrument maker. This enterprising gentleman, it is said, can illuminate the stomach from within by simply causing the patient to swallow an electric light properly insulated in a glass globe. Moreover, this "is double, so that no heat can be communicated to the stomach, and to make it the more sure, the space between the outer and the inner globe is kept supplied with a current of cold water." These seem to be excellent suggestions in their way; but such a stomach full might not prove in every way comfortable. One would have comparatively little objection to swallowing half-a-dozen tallow candles, a few matches, and bits of broken mirrors, with the hope that some fortuitous arrangement would result in the instrument desired; but when he is asked to devour part of an electric battery, he is apt to decline—with thanks. Let Mr. Leiter try again, and perhaps with some improvements he can devise a more tempting and digestible morsel than the present form of his gastroscope.

